# Current News

**Published:** 1905-12

**Authors:** 


### Current News.

### SOUTH DAKOTA STATE BOARD OF DENTAL EXAMINERS.

NOTICE.

THE next meeting of the South Dakota State Board of Dental Examiners will be held at Sioux Falls, S. D., January 16th, 1906, beginning at 1.30 P.M. sharp. All applicants for examination must bring diplomas from reputable dental colleges or affidavit of having been engaged in the practice of dentistry for at least three years immediately preceding said examination. Instruments and materials necessary to do all kinds of operative and prosthetic work will be needed at this examination. Vulcanizer and lathe will be furnished by the Board. All applications must positively be in the hands of the Secretary by January 9th.

G. W. COLLINS, *Secretary,*
Vermillion, S. D.

### AMERICAN SOCIETY OF ORTHODONTISTS.

AT the fifth annual meeting of the American Society of Orthodontists in Chicago, September 28th to 30th, inclusive, the election of officers was announced, as follows: President, Dr. R. Ottolengui, New York, N. Y.; Vice-President, Dr. Herbert A. Pullen, Buffalo, N. Y.; Secretary-Treasurer, Dr. F. S. McKay, St. Louis, Mo.; Member of the Board of Censors, Dr. Milton T. Watson, Detroit, Mich.

F. S. MCKAY,
*Secretary.*

